# Isotopic ratios of uranium and caesium in spherical radioactive caesium-bearing microparticles derived from the Fukushima Dai-ichi Nuclear Power Plant

**DOI:** 10.1038/s41598-020-59933-0

**Published:** 2020-02-24

**Authors:** Yuichi Kurihara, Naoto Takahata, Takaomi D. Yokoyama, Hikaru Miura, Yoshiaki Kon, Tetsuichi Takagi, Shogo Higaki, Noriko Yamaguchi, Yuji Sano, Yoshio Takahashi

**Affiliations:** 1 Department of Earth and Planetary Science, Graduate School of Science, The University of Tokyo (UT), 7-3-1 Hongo, Bunkyo-ku, Tokyo 113-0033 Japan; 2 Atmosphere and Ocean Research Institute, The University of Tokyo (UT), 5-1-5 Kashiwanoha, Kashiwa, Chiba 277-8564 Japan; 3 Geological Survey of Japan, National Institute of Advanced Industrial Science and Technology (AIST), 1-1-1 Higashi, Tsukuba, Ibaraki 305-8567 Japan; 4 Atmospheric and Marine Environmental Sector, Environmental Science Research Laboratory, Central Research Institute of Electric Power Industry (CRIEPI), 1646 Abiko, Abiko, Chiba 270-1194 Japan; 5 Isotope Science Centre, The University of Tokyo (ISC-UT), 2-11-16 Yayoi, Bunkyo-ku, Tokyo 113-0032 Japan; 6 Institute for Agro-environmental Sciences, NARO, 3-1-3 Kannondai, Tsukuba, Ibaraki 305-8604 Japan; 7 Ningyo-toge Environmental Engineering Centre, Japan Atomic Energy Agency (JAEA), 1550 Kamisaibara, Kagamino-cho, Tomata-gun, Okayama 708-0698 Japan

**Keywords:** Environmental impact, Mass spectrometry

## Abstract

Spherical radioactive caesium (Cs)-bearing microparticles (CsMPs) were emitted during the Fukushima Dai-ichi Nuclear Power Plant (FDNPP) accident in March, 2011. The emission source (timing) and formation process of these particles remain unclear. In this study, the isotopic ratios of uranium (^235^U and ^238^U) and caesium (^133^Cs, ^134^Cs, ^135^Cs, and ^137^Cs) isotopes in the five spherical CsMPs (ca. 2 μm in size) sampled at 50 km west of the FDNPP were determined using secondary ion mass spectrometry and laser ablation-ICPMS, respectively. Results showed that the ^235^U/^238^U ratios of CsMPs were homogeneous (1.93 ± 0.03, *N* = 4) and close to those estimated for the fuel cores in units 2 and 3, and that the Cs isotopic ratios of CsMP were identical to those of units 2 and 3. These results indicated that U and Cs in the spherical CsMPs originated exclusively from the fuel melt in the reactors. Based on a thorough review of literatures related to the detailed atmospheric releases of radionuclides, the flow of plumes from the FDNPP reactor units during the accident and the U and Cs isotopic ratio results in this study, we hereby suggest that the spherical CsMPs originate only from the fuel in unit 2 on the night of 14 March to the morning of 15 March. The variation range of the analysed ^235^U/^238^U isotopic ratios for the four spherical particles was extremely narrow. Thus, U may have been homogenised in the source through the formation of fuel melt, which ultimately evaporating and taken into CsMPs in the reactor and was released from the unit 2.

## Introduction

On 11 March 2011, the Great East Japan Earthquake triggered the Fukushima Dai-ichi Nuclear Power Plant (FDNPP) accident. Radioactive materials were released to the atmosphere and have caused various environmental problems^1–4^. Among the six nuclear reactors in the FDNPP, the reactors of units 1–3 were in operation at the time of the earthquake^5^. In the accident, hydrogen explosions occurred in the nuclear reactor buildings of units 1, 3 and 4 (at 15:36 JST on 12 March, 11:01 JST on 14 March and 6:14 JST on 15 March, respectively), but the explosion in unit 4 occurred in the reverse flow of hydrogen generated in unit 3^6^. Unit 4 had been shut down from 2010, and all of the fuel were kept in the spent fuel pool (SFP), suggesting that no radionuclide was emitted from unit 4, whereas units 1 and 3 can be the sources of the radionuclides. Meanwhile, the release of radionuclides from reactor pressure vessel (RPV) or blowout panel in unit 2 was also an important source of the radionuclides^7^. Thus, the three reactors of units 1–3 that were damaged by the accident must be the origins of fission products (FPs), such as radiocaesium and radioiodine, found in the environment^8–11^. Although various investigations and research have been conducted to understand the progress of the accident and the current situation in the three reactor units^12^, the differences among these units in terms of the emission source of radionuclides and the extent of damage and meltdown in each unit are still unclear at present.

In this case, the discovery of Adachi *et al*.^13^ of spherical water-insoluble radioactive Cs-bearing microparticles (CsMPs) with a diameter of several micrometers from the air filter in Tsukuba (170 km southwest of the FDNPP) is important, because CsMPs, which were likely to be emitted directly from the FDNPP reactors as solid materials, may contain various information on the phenomena occurring within the FDNPP reactors during the accident. Adachi *et al*.^13^ and subsequent related studies^14–19^ showed that (i) Cs is present in several mass percent; (ii) silicate glass is the matrix of particles; (iii) other elements that are dissolved in the glass are mostly iron (Fe), zinc (Zn), potassium (K), rubidium (Rb), tin (Sn), chlorine (Cl) and sometimes manganese (Mn) and (lead) Pb and (iv) the particles contain trace amounts of U and FPs. These characteristics of CsMPs that were released from the FDNPP are different from those of radioactive particles (‘hot’ particles) found in the Chernobyl Nuclear Power Plant (ChNPP) accident^20,21^. Additionally, Ono *et al*.^22^ found different types of CsMPs from those mentioned above in the northwestern region near the FDNPP. These particles were estimated to be released from unit 1 based on the comparison with the ^134^Cs/^137^Cs activity ratio of the nuclear fuel at the time of the accident (the ratios of units 1, 2 and 3 were approximately 0.94, 1.08, and 1.05, respectively) calculated with the ORIGEN2 code by Nishihara *et al*.^23^. Satou *et al*.^24^ classified these two types of CsMPs into Type-A and Type-B. This classification is mostly due to difference in the emission sources based on the ^134^Cs/^137^Cs activity ratio (Type-A: unit 2 or 3; Type-B: unit 1). More detailed characteristics of Type-A and Type-B CsMPs are summarised in a recent review manuscript^25^.

Abe *et al*.^14^ applied synchrotron radiation (SR)-based X-ray fluorescence (XRF) and SR-X-ray absorption near-edge structure (XANES) analyses by using micro-X-ray beam to the spherical CsMPs (Type-A) sampled at Tsukuba. Their results showed that uranium (U) and other fissiongenic elements are incorporated into the CsMPs. Kogure *et al*.^17^ and Furuki *et al*.^18^ also detected U and fissiongenic elements in CsMPs (Type-A) by scanning transmission electron microscopy (STEM). U detection is important because U may originate from the nuclear fuel in the FDNPP reactors. However, whether U in CsMPs originate only from the nuclear fuel in the FDNPP or not cannot be ascertained only by the U detection using SR-μ-XRF-XANES analysis. Salbu and Lind^26^ pointed out that the isotopic ratios of transuranium elements are essential to show whether the CsMPs originated from the nuclear fuel. U isotopic ratios, such as ^235^U/^238^U, is primarily important for this purpose, because ^235^U (*T*_1/2_ = 7.038 × 10^8^ years) is enriched relative to ^238^U (*T*_1/2_ = 4.468 × 10^9^ years) in nuclear fuel to cause the chain nuclear fission reaction of ^235^U. Thus, the ^235^U/^238^U ratio, which must deviate from its natural ratio (0.0073), can be the most straightforward evidence to show that the U in the CsMPs originated from nuclear fuel.

Imoto *et al*.^27^ successfully measured ^235^U/^238^U isotopic ratio in two CsMPs found from the same sampling site as Fruki *et al*.^18^ by secondary ion mass spectrometry (SIMS). The isotopic ratios were 0.0296 ± 0.0050 (OTZ3: 2.0 km west–southwest from the FDNPP) and 0.0293 ± 0.0030 (KOI2: 3.4 km south–southwest from the FDNPP), which were consistent with that of enriched nuclear fuel, indicating that the U in the CsMPs was not from natural U. The sampling points for OTZ3 and KOI2 were given in Fig. S4 of Supplementary Information. Ochiai *et al*.^28^ found U dioxides (UO_2_) and debris fragments in CsMPs sampled at the same sampling site as Furuki *et al*.^18^. However, these CsMPs were not spherical unlike the typical CsMPs^13–15,17,19^. Moreover, the Cs concentration as Cs_2_O (in wt.%) and the distribution of constituent elements inside the CsMPs were also different from the typical CsMPs^15,17,19^. Satou *et al*.^24^, Higaki *et al*.^29^ and Yamaguchi *et al*.^30^ have also reported nonspherical CsMPs (Type-A), showing that CsMPs (Type-A) have various shapes and chemical compositions. These particles were sampled within 5 km of the FDNPP (Fig. S4).

In this study, we attempted to measure the ^235^U/^238^U isotopic ratio in the typical and spherical CsMPs (Type-A) with diameters of a few micrometers collected at 50 km west from the FDNPP and to compare the results of the calculated values^23^ and those reported in Imoto *et al*.^27^. NanoSIMS was used to measure the ^235^U/^238^U isotopic ratio, which is essential to analyse the small particles. Caesium isotopic ratios (^135^Cs/^137^Cs, ^135^Cs/^133^Cs, and ^137^Cs/^133^Cs) in the CsMP were also measured to support the results. Although ^135^Cs and ^137^Cs with high yields of 6.5% and 6.2%, respectively, are important FPs, their production processes are different. ^137^Cs is generated in a series of fission chains, whereas ^135^Cs is produced when decay of ^135^Xe is suppressed by ^136^Xe (stable isotope) production because of the neutron capture of ^135^Xe (radioisotope; *T*_1/2_ = 9.14 h) in a series of fission chains. This difference in generation process causes a variation in the ^135^Cs/^137^Cs isotopic ratio depending on the difference in the fuel burn-up degree. Compared with both isotopes, ^134^Cs is scarcely generated by nuclear fission (fission yield = 4.4 × 10^−6^%). ^134^Cs, a FP with high yield (6.8%), is produced by the ^133^Cs neutron capture. The ^135^Cs/^137^Cs ratios in environmental samples related to the FDNPP accident (rainwater, soil and plant samples) have been reported^31–35^. However, given that the reported values are obtained from environmental samples, the influence of contamination by the ChNPP accident^36^ and the global fallout^37^ cannot be completely ruled out. In the present study, laser ablation (LA)-ICPMS was used, because simultaneous analysis of multiple elements is essential for Cs because of possible isobaric interference of barium (Ba) isotopes, and mass interferences of various polymeric ions, such as ^95^Mo^40^Ar^+^, ^97^Mo^40^Ar^+^, ^119^Sn^16^O^+^ and ^121^Sb^16^O^+^. Fast mass scanning by ICP-MS allows us to measure these elements to estimate their interferences on Cs. Moreover, although the typical and spherical CsMPs (Type-A) contain Cs at concentrations ranging from 7 wt.% to 12 wt.% (as Cs_2_O)^17^, the radioactive Cs contents and the stable isotope ^133^Cs (100% natural abundance) are currently unclear. In the present study, ^133^Cs, ^135^Cs (*T*_1/2_ = 2.3 × 10^6^ years) and ^137^Cs (*T*_1/2_ = 30.1 years) in the CsMPs were measured by LA-ICPMS, whereas ^134^Cs (*T*_1/2_ = 2.06 years) and ^137^Cs were measured by gamma-ray spectroscopy, which reveals the content of stable Cs supplied from any source other than the FDNPP. This method also enables the identification of the ratios of various Cs isotopes, including stable and radiocaesium isotopes, facilitating the identification of the reactor units (unit 1, 2 or 3) responsible for radionuclide emission based on the Cs isotopic ratio, which is calculated theoretically as a function of fuel burn-up degree unique to each unit^23^. The comparison of the ^235^U/^238^U isotopic ratios, shape, Cs isotopic ratios, and other information among these studies enable us to estimate the emission source and the production progression of the spherical CsMPs (Type-A).

## Results

### Characteristics of the CsMPs

**Figure 1:**
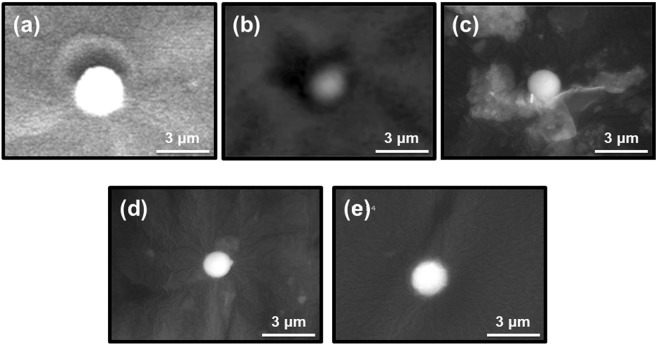
SEM images of five particles separated from the non-woven fabric cloth samples. The particles were placed on Kapton tapes for the SEM-EDS analyses. (**a**–**e**) SEM images of (**a**) particle A (diameter: 2.7 μm), (**b**) particle B (1.8 μm), (**c**) particle C (2.0 μm), (**d**) particle D (1.6 μm), and (**e**) particle E (2.1 μm).

**Figure 2:**
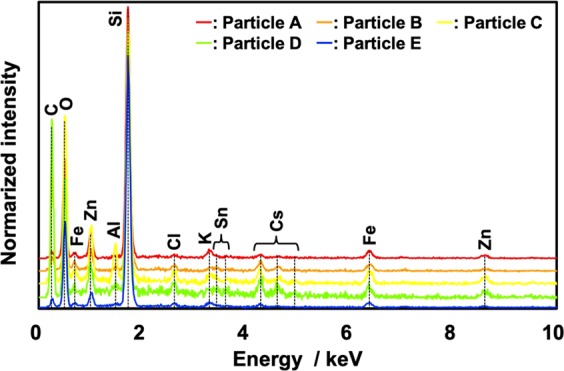
EDS spectra of the five CsMPs.

Figure 1a–e show the scanning electron microscopy (SEM) images of the particles. Particles A (Fig. 1a), B (Fig. 1b) and E (Fig. 1e) were isolated using the wet separation method, whereas particles C (Fig. 1c) and D (Fig. 1d) were separated using the dry separation method^15^. Some images have low resolution, but all particles presented almost spherical shapes with diameters of 1.6–2.7 μm. The characteristics of the five CsMPs are summarised in Table 1. The ^134^Cs and ^137^Cs activities in the particles were 0.73–2.16 and 0.67–1.88 Bq, respectively, as of March 11, 2011. The ^137^Cs activity per unit volume in the particles were consistent with the relationship between ^137^Cs activity and particle volume described by Satou *et al*.^16^. The ^134^Cs/^137^Cs activity ratio with decay-corrected values was approximately 1 for all particles (average = 1.07), and this result agreed with those in previous studies^13–19,24,27–30^. The energy-dispersive X-ray spectrometry (EDS) results of the five particles showed the X-ray peaks of silicon (Si), oxygen (O), Cl, K, Fe, Zn, Sn, Cs and some particles (particles C and D) with aluminium (Al) peak (Fig. 2). The presence of Al in certain particles was considered as secondary adhesion of Al to the particle surface, because Al was scarcely detected inside CsMPs in previous studies^15,17–19,27,28^. The Rb X-ray peak was not identified, because its peak overlapped with the Si peak. As revealed by Kogure *et al*.^17^, the elemental compositions differ slightly among the CsMPs but are basically similar to those in previous studies^13–19,24,27–30^.

**Table 1:** Characteristics of the five CsMPs.

	Particle				
	A	B	C	D	E
Particle size [μm]	2.7	1.8	2.0	1.6	2.1
***Cs isotopes***					
Activity [Bq]^a,b^					
^134^Cs	2.16 ± 0.18	0.91 ± 0.12	1.45 ± 0.15	0.94 ± 0.12	0.73 ± 0.18
^137^Cs	1.88 ± 0.10	0.78 ± 0.06	1.51 ± 0.09	0.94 ± 0.07	0.67 ± 0.10
Activity ratio^a,b^					
^ 134^Cs/^137^Cs	1.15 ± 0.11	1.17 ± 0.18	0.96 ± 0.11	1.00 ± 0.15	1.08 ± 0.12
Isotopic ratio^a^					
^ 135^Cs/^137^Cs	n.m.	n.m.	n.m.	n.m.	0.341 ± 0.016
^ 135^Cs/^133^Cs	n.m.	n.m.	n.m.	n.m.	0.344 ± 0.030
^ 137^Cs/^133^Cs	n.m.	n.m.	n.m.	n.m.	1.01 ± 0.09
***U isotopes***					
Isotopic ratio^b^					
^ 235^U/^238^U (×10^−2^)	1.97 ± 0.11	1.95 ± 0.07	1.90 ± 0.11	1.91 ± 0.05	n.m.

^a^The values are decay-corrected as to March 11, 2011.

^b^Error shows 1σ standard deviation from counting statistics.

^c^Error shows 1σ standard deviation from five replicate measurements of a NIST SRM 610 standard glass.

n.m.: Not measured.

### Isotopic ratio of ^235^U/^238^U in the four CsMPs

**Figure 3:**
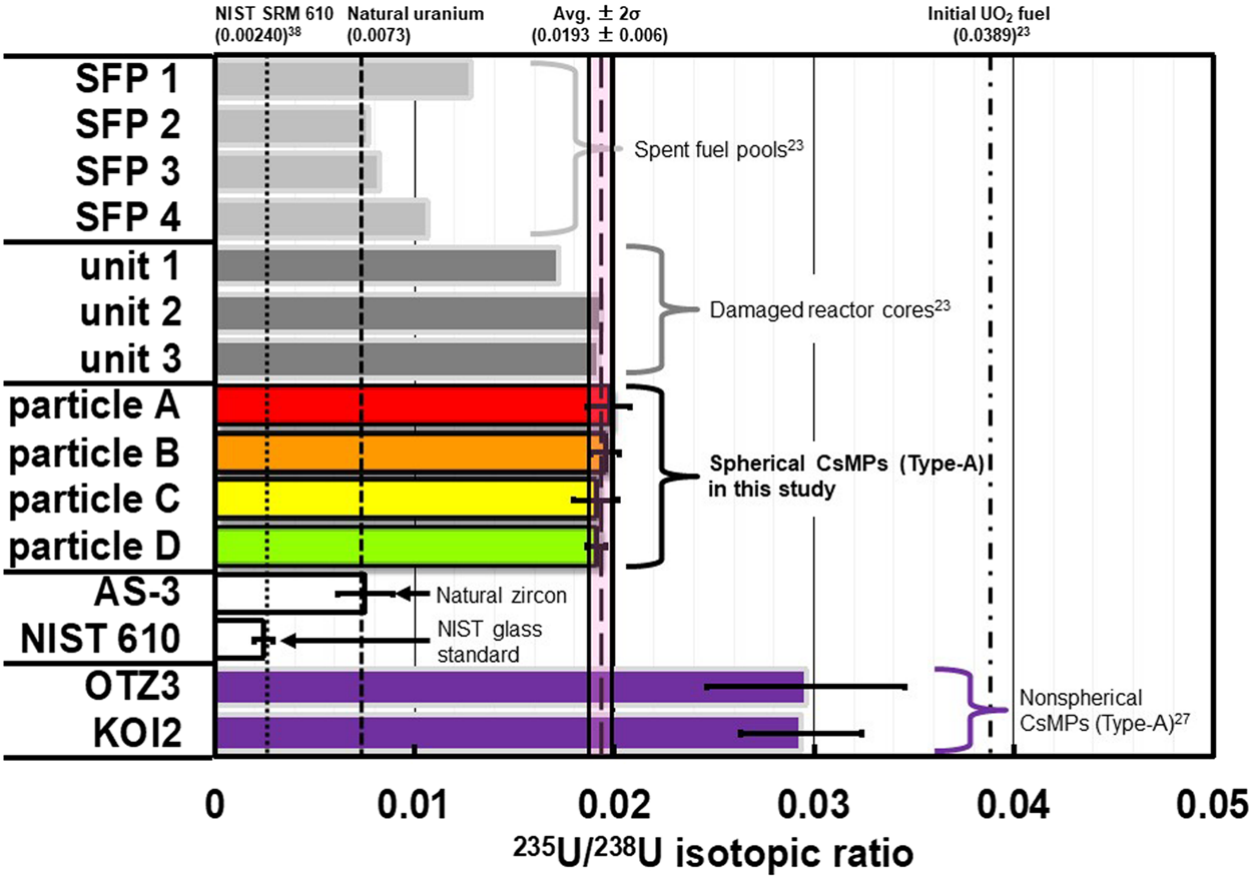
Comparisons of ^235^U/^238^U isotopic ratios observed in the four spherical CsMPs (particles A, B, C and D), NIST SRM 610^38^, natural zircon (AS-3)^46^, the damaged reactor cores (units 1–3)^23^, the spent fuel pools (SFPs 1–4)^23^, the initial UO_2_ fuel^23^, and the nonspherical CsMPs (OTZ3 and KOI2)^27^.

Synthetic silicate glass (NIST SRM 610) and a natural zircon (AS-3) were analyzed as references of ^235^U and ^238^U in the CsMPs, details of which were given in Methods section. The results of ^235^U/^238^U ratio analysis in a NIST SRM 610 and in a natural zircon were 0.00233 ± 0.0010 (2σ) and 0.00737 ± 0.0026 (2σ), respectively. These values coincided with the reference value of the NIST SRM 610 (2.3955 × 10^−3^ ± 4.7 × 10^−7^, depleted U)^38^ and the natural U value (0.0073), respectively, within the error. These results showed that the mass fractionation within the mass spectrometer was negligible. The observed ^235^U/^238^U isotopic ratios, which ranged from 1.90 × 10^−2^ to 1.97 × 10^−2^ (average = 1.93 × 10^−2^), in the four particles (particles A–D) were similar. The observed ^235^U/^238^U ratios were relatively higher than that of natural U and lower than that of enriched U (initial UO_2_ fuel: 0.0389) in the nuclear fuel, as reported by Nishihara *et al*.^23^. The ^235^U/^238^U isotopic ratios in the CsMPs were different from those in the cores of unit 1 and SFP but were very similar to those in the cores of units 2 and 3 (Fig. 3).

### Isotopic ratios of Cs isotopes in the CsMP

**Figure 4:**
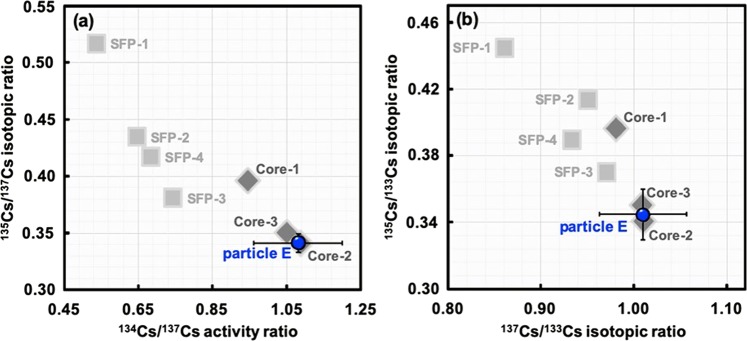
Relationships between (**a**) ^135^Cs/^137^Cs isotopic ratio and ^134^Cs/^137^Cs activity ratio and (**b**) ^135^Cs/^133^Cs and ^137^Cs/^133^Cs isotopic ratios observed in the spherical CsMP (particle E), the damaged reactor cores (units 1–3)^23^, and the spent fuel pools (SFPs 1–4)^23^.

Figure 4a shows the radiocaesium isotopic compositions in the CsMP (particle E) and those estimated for damaged reactor cores (units 1–3) and SFPs 1–4 in the FDNPP. The isotopic composition in the CsMP was different from those in the cores of unit 1 and SFP but was similar to those in the core of unit 2 or 3. As shown in Fig. 4b, the contents of radiocaesium isotopes (^135^Cs and ^137^Cs) relative to the stable ^133^Cs in particle E were also very close to the calculated core values from units 2 and 3 within the error. The ^134^Cs/^133^Cs isotopic ratio calculated from the ^134^Cs/^137^Cs activity ratio also agreed well with those of the cores of units 2 and 3 (0.076 and 0.073, respectively)^23^.

## Discussion

In general, the typical CsMPs (Type-A) from unit 2 or 3 of the FDNPP are extremely small (<10 μm), as suggested by Igarashi *et al*.^25^, and the isolation of CsMPs from many other particles in environmental samples is immensely difficult. Hence, we developed a wet separation method with counting by the NaI scintillation counter, which is described in Sample preparation and Fig. S1. We repeatedly selected one solution sample, which had higher Cs activity, out of two samples in test tubes. After a 30-fold separation, which typically required 5 h, the method can theoretically isolate one CsMP from 1.1 billion (2^30^) particles. The search for radioactive particles by SEM is performed in backscattered electron imaging mode, which allows the identification of CsMPs by searching bright areas that reflect high average atomic number of atoms contained in the particle^20^. As shown in Fig. S2, the wet separation method had significantly less contamination of other particles than the dry separation method. As a result, the search for radioactive particles by SEM became easier. This method (the wet separation method) is a separation method using water, which may affect the conditions of the CsMP surface. However, similar results were obtained in terms of chemical compositions among particles (i) A, B and E and (ii) C and D (Fig. 2 and Table 1). The ^134^Cs/^137^Cs and ^235^U/^238^U isotopic ratios suggested that the alteration caused by the wet separation method was negligible for the chemical and isotopic analyses. Thus, we can conclude that the wet separation method is efficient in separating CsMPs from environmental samples that contain many particles, such as soil.

Previous studies^39–41^ measured the ^235^U/^238^U isotopic ratios of samples contaminated by radiocaesium by the FDNPP accident, such as soil and plant samples from Fukushima Prefecture and vegetable samples from Chiba and Ibaraki Prefectures, which were similar to the natural U isotopic ratio for all the samples. Results revealed that the U in the soil and plant samples was diluted with natural U in the environment. In the present study, particles other than the CsMPs were excluded by the wet separation method prior to NanoSIMS, so that particles A and B were not contaminated with natural U. Moreover, given that almost no U isotopes (^235^U and ^238^U) were detected in the particles surrounding particles C and D separated by dry separation, the influence of contamination by other particles can be ignored in measuring ^235^U/^238^U isotopic ratios in particles C and D. Thus, the U isotopic ratios obtained in the present study has no contribution from Kapton tape or other particles and represents the isotopic composition in these spherical CsMPs.

Direct ^135^Cs and ^137^Cs measurements in the sample containing Ba by mass spectrometry is subject to isobaric interference of Ba isotopes, including ^135^Ba and ^137^Ba, respectively. Similar to previous studies on CsMPs^14,17^, Ba isotope (^138^Ba) was detected also in the present study. In the FDNPP reactor, ^137^Ba and ^138^Ba were produced as FPs, whereas ^135^Ba was not^23^. The ratio between the isotopes deviated from the natural value (natural abundance: ^137^Ba = 11.232%; ^138^Ba = 71.698%). However, the measured intensity of ^138^Ba, which was derived either from nature or from the nuclear reactor, was about 1% of that of (^137^Cs + ^137^Ba) in the particles. This result showed that the isobaric interference of ^137^Ba on ^137^Cs was negligible, because the amount of ^137^Ba was lower than that of ^138^Ba in nature and in the fuel cores (atomic ^137^Ba/^138^Ba ratios of units 1, 2 and 3 were 0.0783, 0.0443 and 0.0442, respectively)^23^. The polymeric interferences of ^95^Mo^40^Ar^+^, ^97^Mo^40^Ar^+^, ^119^Sn^16^O^+^ and ^121^Sb^16^O^+^ should also be taken into consideration when measuring ^135^Cs and ^137^Cs. Molybdenum-95 was not detected in the CsMP (particle E), indicating that the MoAr^+^ interference for ^135^Cs and ^137^Cs was negligible. Tin-118 was not detected by LA-ICPMS, but Sn had been reported to be contained in the spherical CsMPs^13–17,19^. In the present study, Sn was detected slightly by EDS of particle E. This discrepancy was considered to be due to the effect of background (BG) with high Sn, which was reinforced by the low concentration of Sn (2 wt.–3 wt.%) in the spherical CsMPs^15,17^. Meanwhile, ^121^Sb at an intensity of about 9% of that of ^137^Cs was detected in the CsMP. From these results, the ^119^Sn^16^O^+^ and ^121^Sb^16^O^+^ interferences on ^135^Cs and ^137^Cs may occur. However, the intensities of ^118^Sn and ^121^Sb to ^135^Ba and ^137^Cs were only about 0.1% and 2%, respectively, in the BG measurement, indicating that the SnO^+^ and SbO^+^ interferences for Cs isotopes were negligible.

Salbu and Lind^26^ considered that the CsMPs can incorporate a series of stable elements (e.g. Cs, U, Fe and Zn)^42^ present in the environment during formation. However, the various Cs isotopic ratios obtained in the present study were in agreement with those calculated with the ORIGEN2 code^23^. This result indicated that the stable ^133^Cs exclusively originated from the nuclear fuel and that the Cs contribution in any environmental material can be ignored for the Cs isotopes in the CsMPs. These findings revealed that the Cs isotopes in the particle contained radiocaesium (^134^Cs, ^135^Cs and ^137^Cs) and stable Cs (^133^Cs), which were directly emitted from the FDNPP. Importantly, considering the results of U and Cs isotopic ratios, the measured values clearly showed that the U and Cs isotopes in the CsMPs were only supplied from the fuel of unit 2 or 3 without contributions from the natural environment.

We discussed the source of CsMPs (Type-A) based on the values of the U and Cs isotopes in the present study and integrated the interpretation of various information given in literatures here and in the following sections. So far, the detailed atmospheric release of radionuclides and the flow of plumes from the FDNPP reactor units during the accident have been estimated from the air concentration of radionuclides and the air dose rate observed around eastern Japan, the simulation analysis, and comparison with the progress of the accident along time series^8–11^. According to the emission source estimation by Chino *et al*.^10^ based on the analysis of the combination of measured ^134^Cs/^137^Cs depositions on ground surfaces and atmospheric transport and deposition simulations (Worldwide version of SPEEDI: WSPEEDI-II), the major releases of radionuclides from the FDNPP reactor units occurred during 12–21 March 2011, which was divided into the following six periods: (a) the afternoon of 12 March, (b) the night of 14 March to the early morning of 15 March, (c) the morning of 15 March (d) the evening of 15 March to the early morning of 16 March, (e) the morning of 16 March and (f) the midnight of 20 March to the morning of 21 March. The information related to the major releases are summarised in Table S1 ^7,9,10,12^. Determining the emission source of the five CsMPs only by the U and Cs isotopic ratios was difficult to measure in the present study, because the U and Cs isotopic ratios of units 2 and 3 calculated using the ORIGEN2 code were similar (Table S2)^23^. However, based on the estimation of the atmospheric release of radionuclides from each unit by previous studies^8–11^ and on the facts that (i) in Tsukuba^13^, the CsMPs were found in the air filter of 14–15 March and not found in the filter of 20–21 March and that (ii) the releases of radioactive materials from unit 3 during the period (d) were conducted after wet venting^9,10^, the spherical CsMPs analysed in the present study were probably released from unit 2 during the night of 14 March to the morning of 15 March. These periods included the incidents of process (b), (c) and (d) and are summarised in Table S1.

As shown in Fig. 3, the ^235^U/^238^U isotopic ratios in the four spherical CsMPs (Type-A) (approximately 0.019) obtained in the present study were different from the values of the two nonspherical CsMPs (Type-A) (approximately 0.030) measured by Imoto *et al*.^27^. This variance may reflect the differences in the emission timing and formation process of the CsMPs. We hereby suggest that the spherical and nonspherical CsMPs (Type-A) originated from different events based on the integration of various facts reported in the present study and in the other studies below.

The fifth progress report on the FDNPP accident by the Tokyo Electric Power Company (TEPCO)^12^ described that the pressure of the RPV of unit 2 rose from the night of 14 March to the early morning of 15 March, but the safety relief valve (SRV) of the RPV was opened at 21:20 and 23:00 JST on 14 March, and 01:10 JST on 15 March, as a result, the pressure of the RPV decreased. Additionally, the TEPCO’s report^12^ reported that the neutrons that were assumed to be emitted by spontaneous fissions of plutonium (Pu) and curium (Cm) were detected several times in the same period at the main gate of the FDNPP site, which suggested the fuel melting in unit 2 during this period. Therefore, it is likely that the vapor in the RPV containing radionuclides flowed into the drywell (D/W) of unit 2 and began to leak into the environment during this period. Moreover, the TEPCO’s report^12^ showed that the drywell (D/W) pressure of unit 2 was decreased between 07:20 and 11:25 JST on 15 March, the air dose rate of the containment atmospheric monitoring system (CAMS) of the D/W in unit 2 dropped sharply in the morning of 15 March, and steam leaks from the unit 2 blowout panel were confirmed in the morning. Thus, we can assume that radioactive materials from the D/W of unit 2 were released in the morning of 15 March and spread over a wide area.

In TEPCO’s report^12^, because the air dose rate of the CAMS of the D/W in unit 2 rose rapidly in the afternoon of 15 March, the lower head of the RPV was estimated to be damaged, letting a part of molten fuel debris fall to the primary containment vessel (PCV). Afterward, the D/W pressure of unit 2 dropped steeply from 18:00 JST on 15 March to 02:00 JST on 16 March. Meanwhile, Chino *et al*.^10^ estimated that the release during the period (d) created the highest dose rate zone in the western area close to the FDNPP, because the air dose rates at the monitoring posts (MPs) drastically increased at Yamada (4.1 km west-northwest from the FNNPP) at 23:00 JST on 15 March and at Ohno (4.9 km west-southwest from the FNNPP) at 00:00−01:00 JST on 16 March. According to Katata *et al*.^9^, the air dose rate at the MPs also increased at Matsudate (14.2 km south–southwest from the FNNPP) at 03:00 JST on 16 March. Based on this fact, the release of radioactive materials during the period (d) was speculated to affect the south area near the FDNPP. This area within 5 km near the FDNPP overlaps with the sampling sites for the nonspherical CsMPs (Type-A) found by Furuki *et al*.^18^, Satou *et al*.^24^, Imoto *et al*.^27^, Ochiai *et al*.^28^ and Higaki *et al*.^29^. Therefore, the spherical CsMPs analysed in the present study (sampled at 50 km west of the FDNPP) were possibly released in the periods (b) and (c) and were spread over a wide area of eastern Japan (Fig. S3), whereas the nonspherical CsMPs analysed in the previous studies^18,24,27–29^ were released in the period (d) and were deposited in the vicinity of the FDNPP (Fig. S4). During the period (d), it is possible that radioactive materials were also released from unit 3 by wet venting. Wet venting may reduce particulate emissions, but it cannot eliminate them perfectly. Therefore, unit 3 can be another source of the nonspherical CsMPs (Type-A) deposited in the vicinity of the FDNPP.

Yamaguchi *et al*.^15^, Kogure *et al*.^17^ and Okumura *et al*.^19^ analysed the spherical CsMPs collected in the central part of Fukushima Prefecture using transmission electron microscopy (TEM) and STEM with focused-ion-beam (FIB) sample preparation and showed the constituent elements and elemental distributions of the particles. In their results^17^, Si, Fe and Zn existed in a relatively homogeneous distribution inside the spherical CsMPs, whereas Cs, unlike K and Rb, was concentrated in the particle’s rim. Moreover, Okumura *et al*.^19^ found that Fe and Zn, similar to Cs, were concentrated in the spherical particle’s rim. On the formation process of the spherical CsMPs, they proposed that the silicate glass particles were produced as a result of quenching of silicate melt in the atmosphere of the reactor and were generated by contact of molten fuel with the concrete pedestals of the PCV known as molten core concrete interaction (MCCI). They assumed that the concentration of Cs on the surface of the CsMPs occurred because Cs, which was in a gaseous state in the reactor atmosphere, diffused into the glass matrix after formation of the glass particle. Satou *et al*.^16^ and Kogure *et al*.^17^ suggested that another possible source of silica in CsMPs is glass fibre, which covers pipes for water coming in and out of the RPV, in heat insulators. However, Kogure *et al*.^17^ pointed out that if concrete or glass fibre was the source of the silica, it is necessary to explain the absence of Ca and Al, which are the main constituents of concrete and glass fibre, in the CsMPs. Furuki *et al*.^18^ analysed CsMPs, including the nonspherical CsMPs, collected in the area of the vicinity of the FDNPP by STEM coupled with FIB sample preparation and revealed the nanostructure within the CsMPs. They proposed that CsMP formations were associated with the discrete Zn–Fe-oxide nanoparticles, which were embedded through the condensation of SiO vaporised via MCCI. The constituent elements were similar, but the distribution of elements in the nonspherical CsMPs was different from that in the spherical CsMPs. In particular, the concentration of Cs on the particle surface found in the spherical CsMPs had not been observed in the nonspherical CsMPs, suggesting the difference in the formation process of these particles. The nonspherical CsMPs sampled at the same sampling site as Furuki *et al*.^18^ were analysed by Imoto *et al*.^27^ and Ochiai *et al*.^28^ and showed similar characteristics.

From the abovementioned assumptions, although the source of silica is unclear, the spherical CsMPs may have been generated by the interaction between silicate glass and gaseous Cs in the RPV during the night of 14 March to the morning of 15 March (in the periods (b) and (c)). It is likely that nuclear fuel containing U isotopes was almost homogenized and then a small amount of U isotopes vaporized and taken into silicate glass. Meanwhile, the nonspherical CsMPs incorporating UO_2_ and fuel debris (with high ^235^U/^238^U isotopic ratio ≈ 0.030) were possibly generated by the RPV to the PCV transition on the afternoon of 15 March (in the period (d)). According to the TEPCO’s estimation of the present situation of debris^12^, the amount of fuel debris that dropped to the PCV in Unit 2 was smaller than those in units 1 and 3. Therefore, in unit 2, the MCCI is likely to be limited and further investigation of the source of silica in the CsMPs is needed.

The present study, including U and Cs isotopic analysis, can provide certain implications on the emission source (timing) and formation process of CsMPs. Firstly, U and Cs in the spherical CsMPs analysed in the present study (sampled at 50 km west of the FDNPP) were derived only from the fuel in unit 2 during the night of 14 March to the morning of 15 March based on the estimation of the detailed atmospheric releases of radionuclides and the flow of plumes from the FDNPP reactor units during the accident^8–11^. Secondly, the ^235^U/^238^U isotopic ratio of an individual nuclear fuel rod must be heterogeneous, considering the general operation of nuclear reactors. However, the variation range of the analysed ^235^U/^238^U isotopic ratios for the four particles was extremely narrow, suggesting that U was homogenised in the source possibly because of the formation of fuel melt in the RPV of unit 2.

Numerous issues, such as source of silica and incorporation process of various ions into the silicate melt, remain unclear in the formation process of the CsMPs, but the U and Cs isotopic data in this study facilitated an improved understanding of the emission source (timing) of U and Cs and the formation process of the spherical CsMPs emitted during the FDNPP accident. The dissolution of CsMPs in the environment because of weathering^43,44^ and migration to the ocean via rivers^45^ may cause the loss of CsMPs deposited in the environment. An investigation of the spatiotemporal distribution of the U and Cs isotopic ratios of CsMPs (Type-A) in the wide area of eastern Japan is needed to clarify the melting process of nuclear fuel in unit 2.

## Methods

### Sample preparation

Five CsMPs were separated from particles deposited on a non-woven fabric cloth which covered ground in Fukushima Prefecture at 50 km west of the FDNPP during the accident. Details of the cloth sample were described in the previous study^15^. The cloth (20 × 40 cm^2^) was placed on an imaging plate (Fujifilm, BAS-MS 2040) for 20 min, and the areas around high-radioactivity spots were clipped in a 1 cm square obtained by an IP reader (Fujifilm, FLA-9000). The fragment was placed in a 3 mL plastic test tube. Subsequently, 1 mL of Milli-Q (MQ) water was added. The tube was sonicated in an ultrasonic washer bath at room temperature to transfer the radioactive particle(s) from the cloth to the solution. After the ultrasonic treatment, the cloth and test tube were subjected to autoradiography and radioactivity measurement using the NaI scintillation counter (Packard, Cobra 5003), respectively, which showed that the radioactivity was transferred to the solution. After the transfer of the particle to the solution, the test tube was agitated vigorously for 5 s, and the solution was immediately divided into two fractions, which were distributed to two test tubes. The radioactivity of the two tubes was measured with the NaI scintillation counter for a couple of minutes, and the tube with higher radioactivity was selected for further separation of the CsMP(s). MQ water (0.5 mL) was added to the selected tube, and similar separation into two subsequent fractions was conducted as written above. This process, which usually takes less than 10 min, was repeated for a minimum of 30 times to separate the single CsMP from other abundant soil particles completely. The final fraction was reduced to 20–50 μL by decantation and was dropped onto the double-sided Kapton tape (1 cm square) and air-dried. The Kapton tape was placed on an imaging plate for 20 min to confirm the presence of a radioactive spot. Three out of five particles (particles A, B and E) were isolated using wet separation method, whereas two particles (particles C and D) were separated using the dry separation, as described by Yamaguchi *et al*.^15^. The CsMPs were identified finally by SEM (Hitachi S-4500) equipped with EDS (Kevex Sigma). The operating conditions of the instrument included an accelerating voltage of 15 keV and a working distance of 15 mm.

### Measurements of the activity ratio of ^134^Cs/^137^Cs in the five CsMPs by gamma-ray spectrometry

Caesium-134 and ^137^Cs activities in the identified CsMPs were measured by nondestructive gamma-ray spectrometry. Gamma-ray measurements were conducted at NARO and ISC-UT. Gamma-ray spectrometers were calibrated using small sources (NARO: 5 mm square; ISC-UT: 1 mm square) made from ^134^Cs and ^137^Cs standard solutions received from Japan Radioisotope Association, which were calibrated by Japan Calibration Service System. The radioactivity values of the small source in NARO were 424.7 and 375.5 Bq for ^134^Cs and ^137^Cs, respectively, as of August 4, 2014, whereas those prepared in ISC-UT were 0.182 and 1.40 Bq for ^134^Cs and ^137^Cs, respectively, as of November 25, 2016. Gamma-ray measurement time ranged from 3,600 s to 5,400 s for the particles used for the NanoSIMS analysis and 100,000 s for the particle used for the LA-ICPMS analysis. The ^134^Cs and ^137^Cs activities in the particles were determined using gamma-rays at 604.7 and 661.7 keV, respectively.

### Measurements of isotopic ratio of ^235^U/^238^U in the four CsMPs by NanoSIMS

Uranium isotopic ratio of ^235^U/^238^U was determined using the Cameca NanoSIMS NS50 instrument from the Atmosphere and Ocean Research Institute, UT. After gamma-ray measurement, four samples were coated with gold (Au) to prevent charge-up during SIMS analysis. The Au-coated samples were evacuated in an air-lock system overnight and introduced into a sample stage in the analysis chamber of NanoSIMS. Synthetic silicate glass (NIST SRM 610)^38^ and a natural zircon (AS-3)^46^ were analyzed for the calibration of ^235^U and ^238^U in the CsMPs. The measured particles were a few micrometers in size, as shown in Fig. 1 and Table 1. Three detectors (electron multipliers) were used to analyze the ^56^Fe^+^, ^235^U^+^, and ^238^U^+^ (or ^235^U^16^O^+^ and ^238^U^16^O^+^) ions simultaneously by ion imaging using a focused (<0.5 μm) primary O^-^ beam (current: 40 pA). The primary ion beam was scanned over the 2 × 2 μm^2^ to 5 × 5 μm^2^ areas. The scan was repeated for 20–40 cycles. The total time required for the analysis was approximately 10–20 min for each particle. The U isotopic ratio was calculated by integrating the signals for each particle. The total ^235^U ion counts were typically several hundreds. Moreover, the BG level determined by the same experimental procedure without the particle was less than 2% of the sample levels. After BG correction, the sensitivity difference between the detectors for ^235^U and ^238^U was corrected. Isotopic composition was calibrated using the AS-3 zircon. We confirmed that the analytical technique does not induce artificial isotope anomalies by isotope imaging of the NIST standard glass.

### Measurements of isotopic ratios of Cs isotopes in the CsMP by LA-ICPMS

The ^135^Cs/^137^Cs, ^135^Cs/^133^Cs, and ^137^Cs/^133^Cs isotopic ratios were determined using LA-ICPMS. The LA-ICPMS system was equipped with the UV-femtosecond LA system Cyber Laser IFRIT (Tokyo, Japan) and Agilent Technologies 7500 cx (Santa Clara, CA, USA). The LA system based on a 230 fs TiS system was operated at a wavelength, repetition, ablation pit size, fluence and total ablation time of 260 nm, 10 Hz, 10 μm, 3.6 J cm^−2^ and 40 s, respectively. The operational conditions of the ICPMS instrument were optimized to maximize the signal intensity of the ^133^Cs signal obtained by NIST SRM 610 standard glass^38^. The LA diameter used was larger than the size of the CsMP. Thus, the CsMP was totally ablated by the 10 μm laser beam. The ^135^Ba/^137^Ba, ^135^Ba/^133^Cs and ^137^Ba/^133^Cs isotopic ratios in the NIST standard glass^38^ were measured to confirm the mass discrimination and interference effects on Cs isotopic ratios measured in the CsMPs. The BG signal used for its corrections were obtained from blank point ablation (*n* = 5) of the Kapton tape, which was used to affix the CsMP. Additionally, ^95^Mo, ^118^Sn, and ^121^Sn were simultaneously analyzed in the measurement of BG and sample in order to evaluate the interference of Ba isotopes and the polymeric interferences of ^95^Mo^40^Ar^+^, ^97^Mo^40^Ar^+^, ^119^Sn^16^O^+^, and ^121^Sb^16^O^+^. The Cs isotopic ratio errors of ^135^Cs/^137^Cs, ^135^Cs/^133^Cs, and ^137^Cs/^133^Cs in the CsMPs were estimated from five replicate measurements of ^133^Cs, ^135^Ba, and ^137^Ba in the NIST standard glass.

## Supplementary information

Supplementary information.

## Data Availability

The data that support the findings of this study are available upon request from the corresponding author [Y.T.].
