# Supplementary material for: Isotopic ratios of uranium and caesium in spherical radioactive caesium-bearing microparticles derived from the Fukushima Dai-ichi Nuclear Power Plant
Source: Sci Rep. 2020 Feb 24;10:3281. doi: 10.1038/s41598-020-59933-0 (PMC7039901; doi:10.1038/s41598-020-59933-0)
Supplement: Supplementary file 1 — Supplementary information. [file 41598_2020_59933_MOESM1_ESM.pdf]

Supplementary Information for
Isotopic ratios of uranium and caesium in spherical radioactive caesium-bearing microparticles derived from the Fukushima Dai-ichi Nuclear Power Plant

Yuichi Kurihara[1,7], Naoto Takahata[2], Takaomi D. Yokoyama[3], Hikaru Miura[4], Yoshiaki Kon[3], Tetsuichi Takagi[3], Shogo Higaki[5], Noriko Yamaguchi[6], Yuji Sano[2], and Yoshio Takahashi[1,*]

[1] Department of Earth and Planetary Science, Graduate School of Science, The University of Tokyo (UT), 7-3-1 Hongo, Bunkyo-ku, Tokyo 113-0033, Japan

[2] Atmosphere and Ocean Research Institute, The University of Tokyo (UT), 5-1-5 Kashiwanoha, Kashiwa, Chiba 277-8564, Japan

[3] Geological Survey of Japan, National Institute of Advanced Industrial Science and Technology (AIST), 1-1-1 Higashi, Tsukuba, Ibaraki 305-8567, Japan

[4] Atmospheric and Marine Environmental Sector, Environmental Science Research Laboratory, Central Research Institute of Electric Power Industry (CRICPI), 1646 Abiko, Abiko, Chiba 270-1194, Japan

[5] Isotope Science Centre, The University of Tokyo (ISC-UT), 2-11-16 Yayoi, Bunkyo-ku, Tokyo 113-0032, Japan

[6] Institute for Agro-environmental Sciences, NARO, 3-1-3 Kannondai, Tsukuba, Ibaraki 305-8604, Japan

[7] Ningyo-toge Environmental Engineering Centre, Japan Atomic Energy Agency (JAEA), 1550 Kamisaibara, Kagamino-cho, Tomata-gun, Okayama 708-0698, Japan

*Corresponding author: Yoshio Takahashi (ytakaha@eps.s.u-tokyo.ac.jp)

Table S1 Summary of the major releases of radionuclides from the FD1NPP reactor units in the six periods of 12−21 March 2011[7,9,10,12]

Release	Period	Incident	Monitoring post* (distance and direction from the FD1NPP)	Plume direction (WSPEEDI-II calculation)
(a)	From 15:30 to 16:00 JST on12 March, 2011	unit 1: Hydrogen explosion occurred the building at 15:36 JST on 12 March.	Shinzan (3.9 km north-northwest), Namie (8.6 km north-northwest), Kiyohashi (8.2 km north)	The plume flowed toward the north-northwest direction.
(b)	From 21:00 JST on 14 March, 2011 to 02:00 JST on 15 March, 2011	unit 2: The pressure of the reactor pressure vessel (RPV) rose three times from the night of 14 March to the early morning of 15 March. The safety relief valve (SRV) of the RPV was opened three times at 21:20 and 23:00 JST on 14 March, and 01:10 JST on 15 March, as a result, the pressure of the RPV decreased.<br>Neutrons were detected several times near the main gate from 21:00 JST on 14 March to 01:40 JST on 15 March.	FD2NPP (11.4 km south), Kitaibaraki (80 km south), JAEA-Tokai (100 km south)	The plume flowed toward the south-southwest direction, and then moved westward and wet deposition at the mountainous regions of Gunma and Tochigi Prefectures and the central part of Fukushima Prefecture.
(c)	From 07:00 to 11:00 JST on 15 March, 2011	unit 2: The drywell (D/W) pressure was decreased between 07:20 and 11:25 JST on 15 March, the air dose rate of the containment atmospheric monitoring system (CAMS) of the D/W dropped sharply in the morning of 15 March and steam leaks from the building's blowout panel were confirmed in the morning.	Main gate located on the south side of the damaged reactors, Ohno (4.9 km west-southwest), Yamada (4.1 km west-northwest), Shirakawa City (81 km west-southwest), Koriyama City (58 km west), Fukushima City (67.2 km northwest), Iitate Village (38.9 km northwest)	The plume flowed toward the southwest direction, and reached the central area of Fukushima Prefecture, including Shirakawa City at 81 km west and Koriyama City at 58 km west from the FDNPP, and then gradually drifted northward and encountered the rain band in the early evening of the day in the northwest area of the FDNPP including Fukushima City and Iitate Village.
(d)	From 16:00 JST on 15 March, 2011 to 01:00 JST on 16 March, 2011	unit 2: The air dose rate of the CAMS of the D/W rose rapidly from 13:00 to 16:10 JST on 15 March and the D/W pressure dropped steeply from 18:00 JST on 15 March to 02:00 JST on 16 March.<br>unit 3: The wet venting was conducted at 16:05 JST on 15 March, corresponding to the decline in the D/W pressure. Afterward, wet venting was carried out at Unit 3 several times, and the decline in the D/W pressure finally stopped around 06:00 on 16 March.	Namie (8.6 km north-northwest), Yamada (4.1 km west-northwest), Ohno (4.9 km west-southwest), Matsudate (14.2 km south-southwest)	The plume flowed toward the west-northwest direction, and then moved to the northwest during the evening of 15 March, and around the midnight, the flow returned toward the west and then finally toward the south area of the FDNPP over the early morning of 16 March.
(e)	From 09:00 to 11:00 JST on 16 March, 2011	unit 3: The D/W pressure was decreased from 09:00 to 11:00 JST on 16 March. In addition, white smoke from the building was observed at 08:30 JST on 16 March.	Ohno (4.9 km west-southwest), Matsudate (14.2 km south-southwest)	The plume flowed toward the Pacific in the morning, returned to the coastal area around noon, and then reversed back toward the Pacific once again before finally moving deeply into inland areas.
(f)	From 00:00 JST on 20 March, 2011 to 06:00 JST on 21 March, 2011	unit 3: The suppression chamber (S/C) venting was carried out at 11:25 JST on 20 March until the decrease of the DW pressure.	Tokyo-Sinjuku (225 km south-southwest), Chiba City (221 km south)	The plume flowed southward into the Pacific, subsequently migrated to a landfall position from the Pacific during the morning of 20 March, and then moved to the north direction, and finally the flow returned the southward

***: The monitoring post with drastically increased in air dose rate during and/or after the release period.**

Table S2 Estimated results of uranium and cesium isotopic compositions in the cores of units 1-3 and in the spent fuel pools (SFPs)[23]

Ratio	Core						
	unit 1	unit 2	unit 3	SFP-1	SFP-2	SFP-3	SFP-4
$^{235}U/^{238}U$ isotopic ratio ($\times 10^{-2}$)	1.72	1.93	1.92	1.28	0.769	0.823	1.06
$^{134}Cs/^{137}Cs$ activity ratio	0.941	1.08	1.05	0.538	0.648	0.744	0.686
$^{135}Cs/^{137}Cs$ isotopic ratio	0.396	0.341	0.350	0.516	0.435	0.381	0.417
$^{135}Cs/^{133}Cs$ isotopic ratio	0.388	0.344	0.353	0.445	0.413	0.370	0.389
$^{137}Cs/^{133}Cs$ isotopic ratio	0.981	1.01_1	1.00_9	0.861	0.957	0.971	0.934
$^{134}Cs/^{133}Cs$ isotopic ratio	0.064	0.076	0.073	0.032	0.042	0.050	0.044

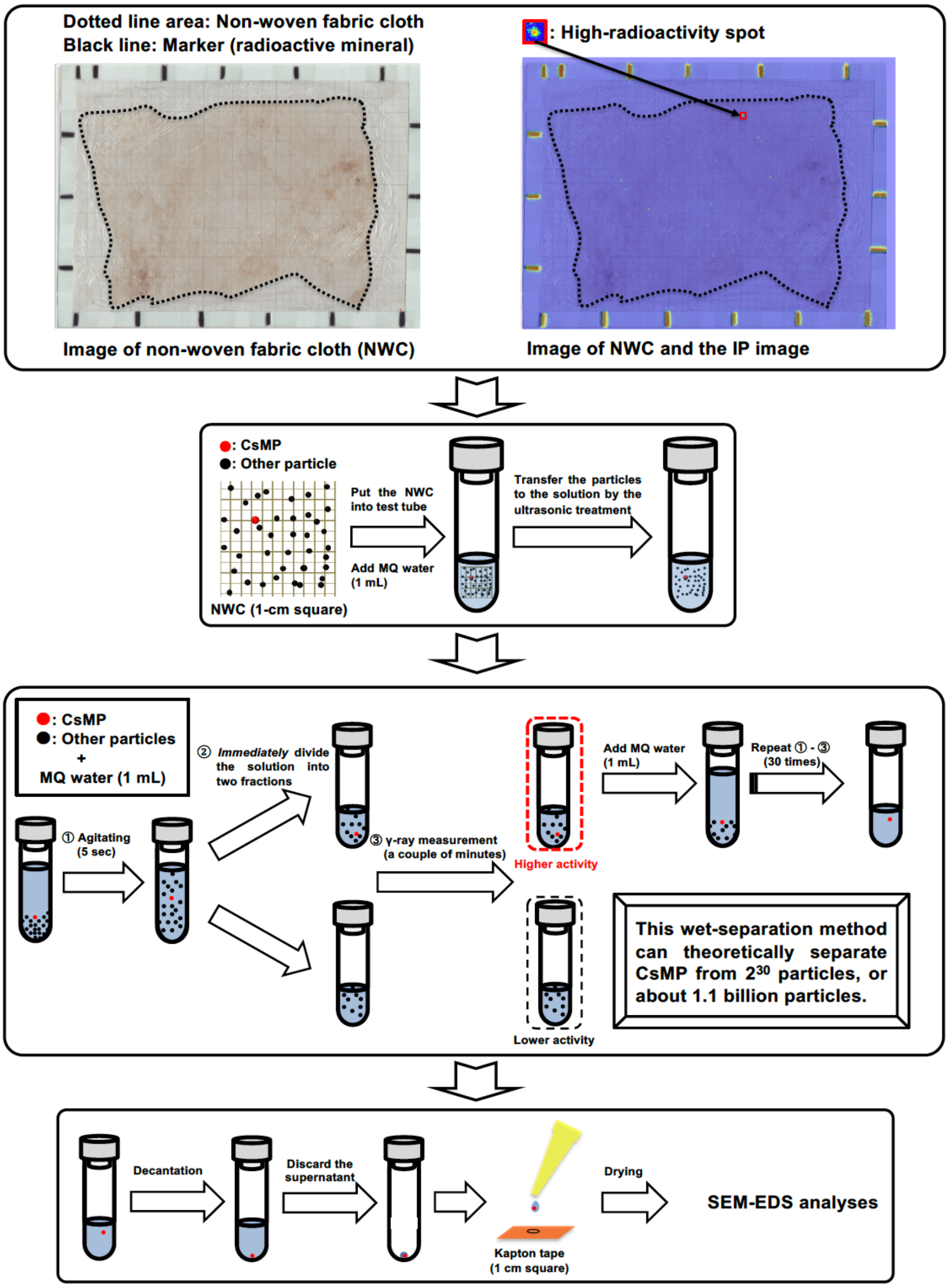

Figure S1 Wet separation method.

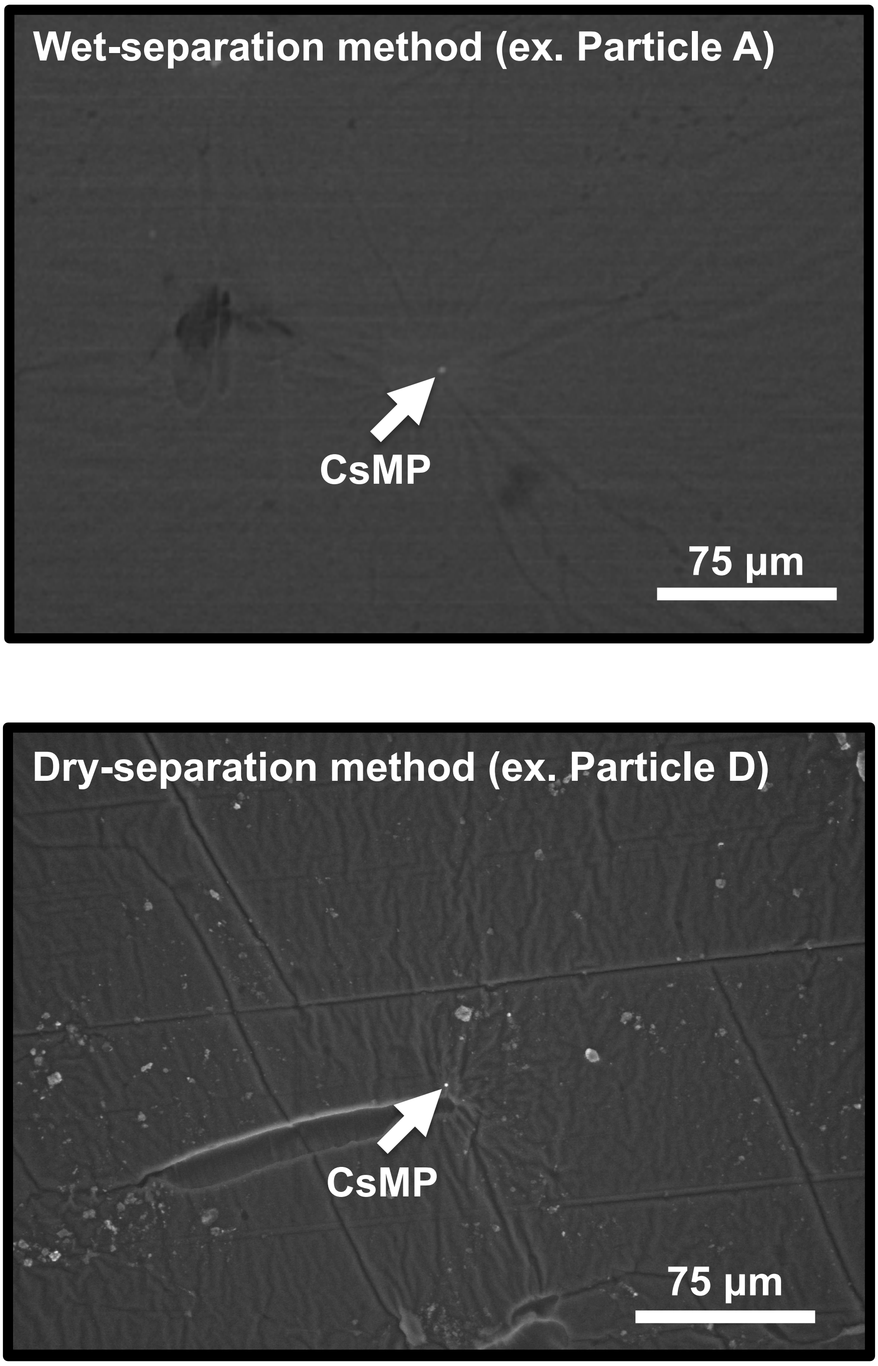

Figure S2 Backscattered electron images of Kapton tape containing the particles after the two separation methods.

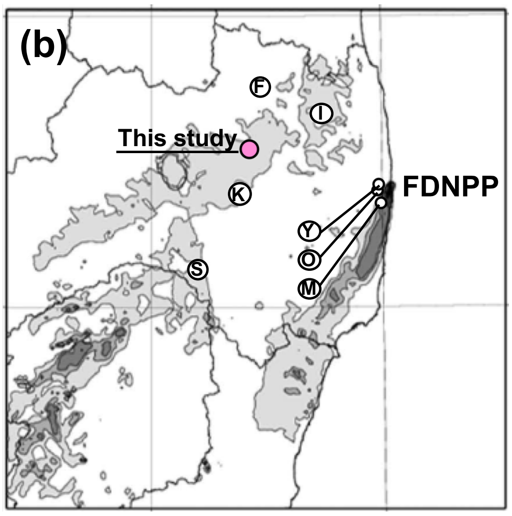

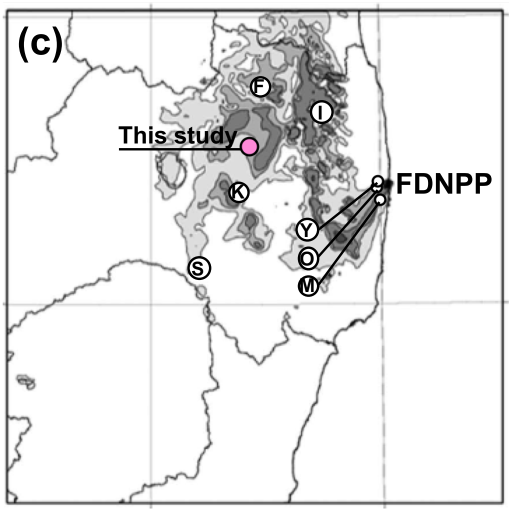

b) Release from the night of 14 March to the early morning of 15 March

c) Release in the morning of 15 March

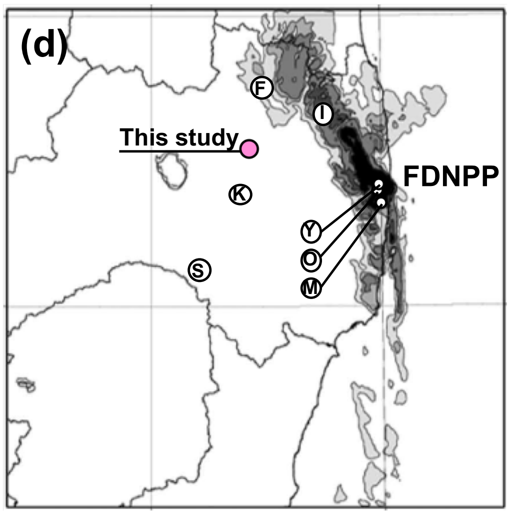

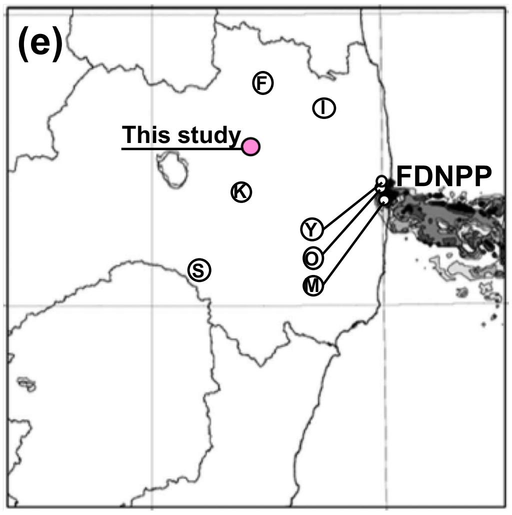

d) Release from the evening of 15 March to the early morning of 16 March

e) Release in the morning of 16 March

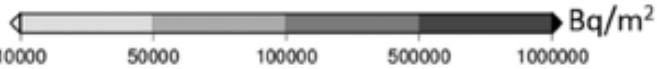

Monitoring posts

(S): Shirakawa City (81 km west-southwest) (K): Koriyama City (58 km west) (F): Fukushima City (67.2 northwest)

(I): Iitate Village (38.9 km northwest) (Y): Yamada (4.1 km west-northwest) (O): Ohno (4.9 west-southwest)

(M): Matsudate (14.2 south-southwest)

Figure S3 Deposition patterns of ^{137}Cs over Fukushima Prefecture calculated by WSPEEDI-II for the periods (b) to (e),* the locations of the monitoring posts, and the sampling point in this study. *: The deposition patterns are from Chino et al. (2016).[10] **: The locations of the MPs are based on Katata et al. (2015).[9]**

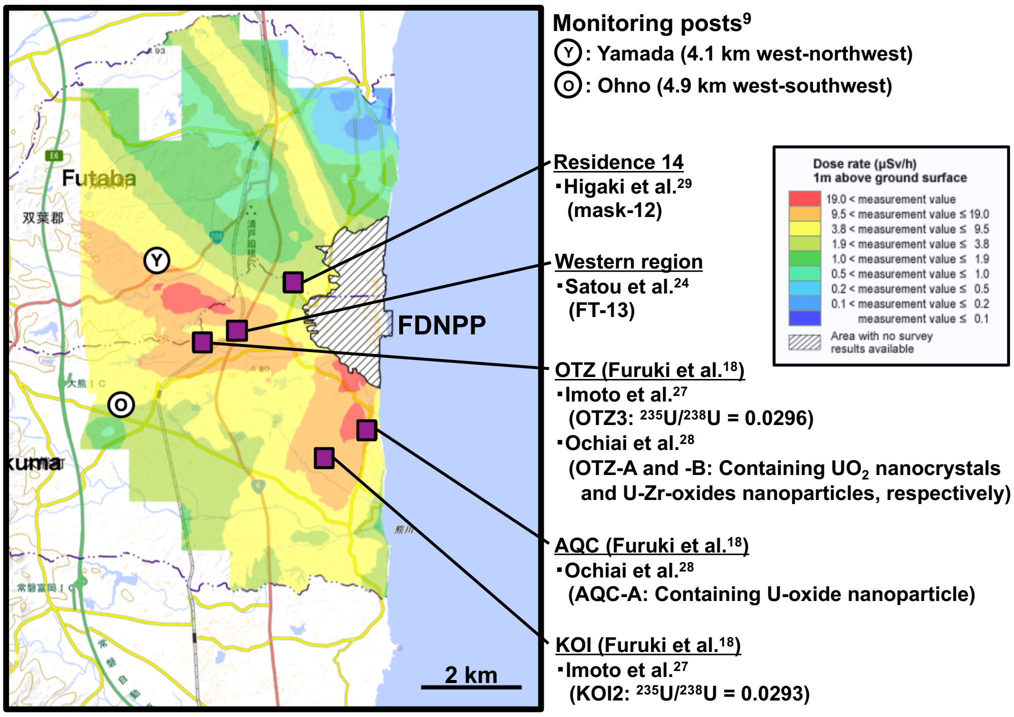

Figure S4 Distribution of the non-spherical CsMPs found in the vicinity of the FDNPP.
